# Enlightenment of Growth Plate Regeneration Based on Cartilage Repair Theory: A Review

**DOI:** 10.3389/fbioe.2021.654087

**Published:** 2021-06-03

**Authors:** Xianggang Wang, Zuhao Li, Chenyu Wang, Haotian Bai, Zhonghan Wang, Yuzhe Liu, Yirui Bao, Ming Ren, He Liu, Jincheng Wang

**Affiliations:** ^1^Orthopaedic Medical Center, The Second Hospital of Jilin University, Changchun, China; ^2^Orthopaedic Research Institute of Jilin Province, Changchun, China; ^3^Department of Plastic and Reconstructive Surgery, The First Hospital of Jilin University, Changchun, China; ^4^Department of Orthopedics, Chinese PLA 965 Hospital, Jilin, China

**Keywords:** growth plate, cartilage tissue engineering, scaffold, bone marrow mesenchymal stem cells, three-dimensional printing

## Abstract

The growth plate (GP) is a cartilaginous region situated between the epiphysis and metaphysis at the end of the immature long bone, which is susceptible to mechanical damage because of its vulnerable structure. Due to the limited regeneration ability of the GP, current clinical treatment strategies (e.g., bone bridge resection and fat engraftment) always result in bone bridge formation, which will cause length discrepancy and angular deformity, thus making satisfactory outcomes difficult to achieve. The introduction of cartilage repair theory and cartilage tissue engineering technology may encourage novel therapeutic approaches for GP repair using tissue engineered GPs, including biocompatible scaffolds incorporated with appropriate seed cells and growth factors. In this review, we summarize the physiological structure of GPs, the pathological process, and repair phases of GP injuries, placing greater emphasis on advanced tissue engineering strategies for GP repair. Furthermore, we also propose that three-dimensional printing technology will play a significant role in this field in the future given its advantage of bionic replication of complex structures. We predict that tissue engineering strategies will offer a significant alternative to the management of GP injuries.

## Introduction

The growth plate (GP), or the physis, is a cartilaginous region situated between the epiphysis and metaphysis at the end of immature long bones. It acts as the primary center for longitudinal growth in children’s long bones (Kronenberg, 2003; Mackie et al., 2011). Being cartilaginous, the GP is the weakest region in the pediatric skeleton, and is vulnerable to injuries including infections, fractures, bone tumors, and iatrogenic damage. The most common sites of GP injuries are the ankle, the distal femur, and the distal radius (MacIntyre and Dewan, 2016). According to the epidemiological data, GP injuries account for 15–30% among all pediatric skeletal injuries (Shen et al., 2019). The major problem with GP injuries is that the injured GP cartilage will be replaced by undesirable bony tissue, forming a bone bridge, which may cause length discrepancies and angular deformities (Gigante and Martinez, 2019). This result can be detrimental to children who are still in the growth phase. Current clinical treatments often include the use of interpositional materials as fillers at the site of the defect after resection of the bone bridge, these materials include autogenous fat, muscle, and cement. Conversely, when the bone bridge occupies less than 50% of the GP, it will require surgical intervention to resect the bone bridge to insert different interpositional materials including fat, bone wax, muscle, or polymeric silicone materials. However, the clinical success of this surgery is less than 35%, as currently available interpositional materials do not integrate well with host tissues and often lead to subsequent complications. Conversely, when the bone bridge occupies over 50% of the GP, it will necessitate corrective surgeries and limb lengthening procedures in clinic. Similarly, outcomes are unsatisfactory (Ladenhauf et al., 2020). Unfortunately, clinical efforts will lead to secondary damage or result in the recurrence of bone bridge formation (Shaw et al., 2018). It is critical to identify new approaches to prevent bone bridge formation and to promote tissue regeneration.

In recent years, due to the introduction of cartilage repair theory, cartilage tissue engineering has been considered a potential alternative treatment for GP injuries (Diaz-Payno et al., 2020). This technology mainly involves seed cells, growth factors, and scaffolds. The seed cells are expanded *in vitro* and are implanted into the scaffolds to form a cell-based scaffold (Zhao et al., 2019). Although seed cells are influenced by the microenvironment *in situ*, growth factors are still critical to inducing cells to differentiate into desired lineages. Scaffolds fabricated by biocompatible and biodegradable materials, with three-dimensional (3D) structures and suitable mechanical strength, can serve as a substitute for GP defects (Li et al., 2017). After being implanted in the GP defects, the scaffold is degraded gradually during the formation of the new cartilage tissue (Liu J. Y. et al., 2020). Although many studies have been carried out and have achieved good results, there is no consensus on the most suitable materials, seed cells, or growth factors (Erickson et al., 2018). Hence, cartilage tissue engineering still requires more intensive studies in the future.

In this review, we will summarize the histological structure of the GP and pathological processes occurring during bone bridge formation. We will review the progress achieved in tissue engineering for treatment of GP injuries, the challenges in clinical application, and the prospect for the future development will also be analyzed (Scheme 1).

**SCHEME:**
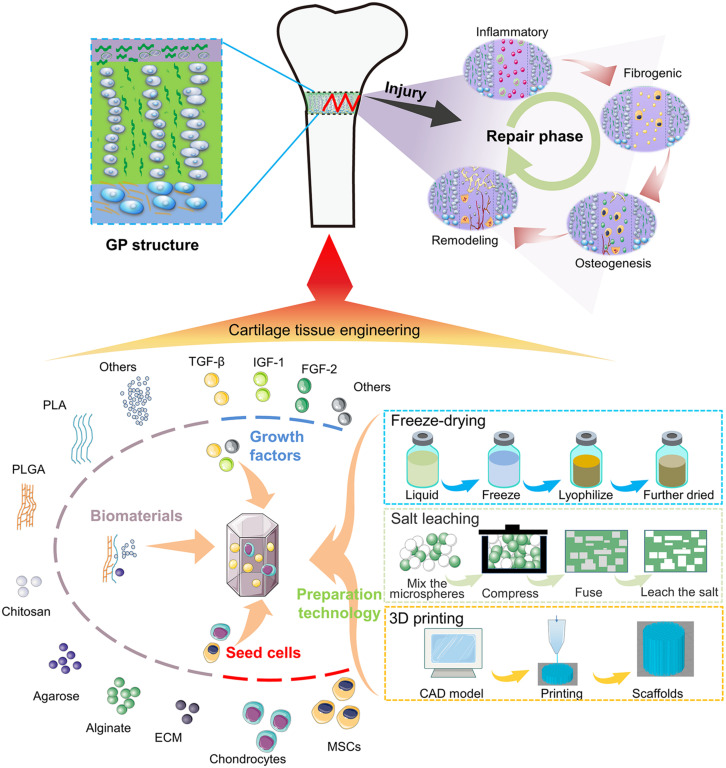
Illustration of the bone bridge formation and the treatment of the GP injuries using tissue engineering.

## The Mechanism of the GP Damage

### Physiological Characteristics of the GP

#### The Histological Structure of the GP

**FIGURE 1:**
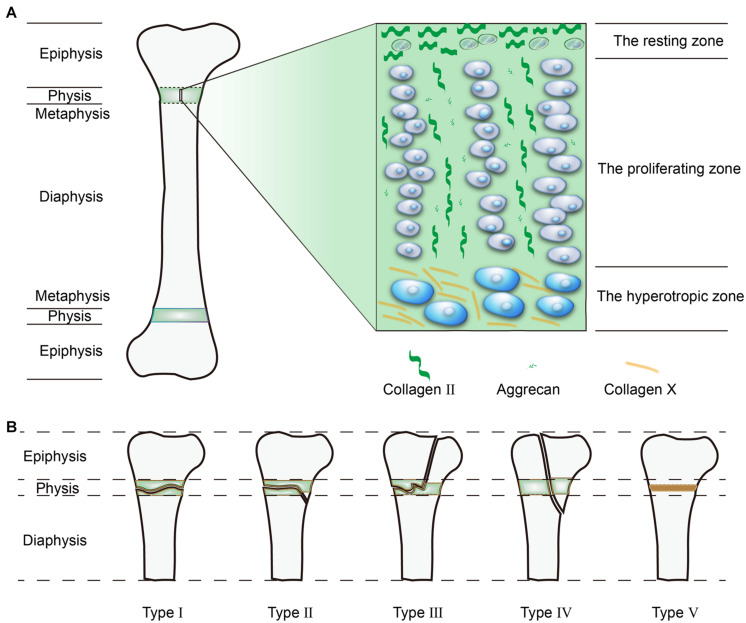
**(A)** Structure of the GPs. **(B)** The Salter-Harris (SH) Classification System.

The differentiation stages of chondrocytes divide the GP into three distinct zones, from the epiphysis to metaphysis: the resting zone, proliferation zone, and hypertrophic zone (Liu et al., 2019; Figure 1A). Because of the cell types and locations, the composition of the extracellular matrix (ECM) also differs in terms of mechanical strength. Furthermore, the proportions between the three zones also differs among species.

Adjacent to the epiphysis, the resting zone forms a reservoir of stem cells or progenitor cells for chondrocytes in the proliferative zone. Each cell can differentiate into chondrocytes and forms a cell column parallel to the axis of long bones (Newton et al., 2019). Cells located here also secrete parathyroid hormone-related protein (PTHrP), which modulates GP homeostasis by interacting with a growth factor called the Indian hedgehog (Ihh) secreted by hypertrophic chondrocytes (Mizuhashi et al., 2018). PTHrP mainly preserves the population of resting cells and promotes chondrocyte proliferation at the upper region of the GPs. Conversely, Ihh directly antagonizes PTHrP signaling and promotes chondrocyte hypertrophy at the lower region of the GPs (Lee D. et al., 2019). Although these resting cells possess the ability to produce cartilaginous matrix, they tend to remain inactive with lower collagen II (Col II) and proteoglycan production. The main composition of the ECM in this zone is horizontally aligned Col II (Mizuhashi et al., 2019).

Located adjacent to the resting zone, the proliferative zone is vital for cellular division and matrix production, it contains vertically arranged chondrocytes. These longitudinal columns of chondrocytes are separated by the cartilage matrix surrounding them. The ECM is mainly produced in this zone and is enriched in vertically aligned Col II and aggrecan (Lui et al., 2014; Shaw et al., 2018).

In addition, regulated by different growth factors, including bone morphogenic protein (BMP), fibroblast growth factor (FGF), insulin-like growth factor-1 (IGF-1), and tumor necrosis factor (TNF), chondrocytes in the hypertrophic zone stop proliferating, and swell in size (Chung and Xian, 2014). By analyzing the relationship between different parameters and bone growth, the enlargement of chondrocytes is mostly associated with the longitude growth (44–59%) (Cooper et al., 2013). The hypertrophic zone is mainly associated with matrix mineralization, where the ECM is mostly composed of Col X (Pazzaglia et al., 2020). Under the influence of low oxygen tension and in the presence of vascular endothelial growth factor (VEGF), the hypertrophic zone allows blood vessels invasion from the metaphysis. The vessels bring osteoblasts, osteoclasts, and mineralized cartilage-resorptive cells to the zone, and thus convert the mineralized matrix into a bone trabecular-like metaphysis (Cheng et al., 2019). As for hypertrophic chondrocytes in this zone, hypertrophic cells were originally believed to be the final state of chondrocyte differentiation. However, recent fate-mapping studies have altered this view since some hypertrophic chondrocytes differentiate into osteoblasts or progenitor cells instead of undergoing apoptosis. Thus, it is tempting to investigate how resting stem cells establish the fate of hypertrophic cells (Yang et al., 2014; Zhou et al., 2014; Park et al., 2015).

#### Mechanical Properties of the GP

In the past, studies were limited by ethical concerns and access to materials, and only a few experiments tested the mechanical properties of human GPs. Most studies were based on animal testing, including piglets (Shen et al., 2019), bovine (Cohen et al., 1992), and sheep models (Celarek et al., 2014). In general, for 10-year-old children, with a loading rate of 0.003 s^–1^, the mean human ultimate stress is 0.98 ± 0.29 MPa, the mean human ultimate strain is 31 ± 7%, and the mean human tangent modulus is 4.26 ± 1.22 MPa (Williams et al., 2001). Animal studies have demonstrated that the lateral region of the proximal tibial GPs was stronger than the central region and the ultimate tensile strength was similar in different parts of the body. However, the tensile strength is largely affected by the GP thickness. For example, the capital femoral GPs in humans is twice as thick as the bovine proximal tibial GPs, but the tensile strength is only half bovine GPs (Cohen et al., 1992; Williams et al., 2001; Sylvestre et al., 2007). Thus, the GP tends to be weaken as it thickens.

It is important to imitate the natural mechanical properties of GPs in order to successfully engineer cartilage tissue scaffolds. However, to date these characteristics have not been clearly elucidated and additional in-depth studies are required.

Biomechanics plays important role in GP formation and function as well. By varying the loading frequency, amplitude, or duration, it is shown to affect the height, morphology, gene expression, and matrix mineralization of the GP (D’Andrea et al., 2020). Stokes et al. (2007) demonstrated that intermittent static loading increased GP height while persistent static loading decreased it. As for chondrocyte count in proliferative zone, it is shown to increase in tension, but decrease under compression using persistent static load. Similarly, persistent static tension is reported to stimulate hypertrophic zone height while compression is found to reduce it (Killion et al., 2017). Except for the mechanical loads on GPs, the mechanical properties of biomaterials used in scaffolds are reported to affect chondrogenesis as well. For instance, cells exposed to stiffer substrates showed a more organized cytoskeleton and faster proliferation rate. Moreover, on softer substrates, cells tended to migrate faster than stiffer one (Ghosh et al., 2007). In conclusion, intermittent tension lading with stiffer scaffolds may benefit GP reconstruction. It is important to figure out the effects of different forms of mechanical stimulation on engineered GPs and to find the optimized stimulus for GP reconstruction. Understanding the effects of mechanical stimulation may help find targets for mechanical strategies for GP repair as well.

#### Differences Between GP and Other Cartilages

The key for GP regeneration is to reconstruct the gradient differentiation states of chondrocytes in a columnar structure. Enlighten by cartilage regeneration, in order to promote GP regeneration based on cartilage repair theory, it is important to dig up the difference and similarity between GP and other cartilage, especially with articular cartilage.

In cartilage, the GP is the most unique. Among all cartilage inhuman, articular cartilage is the most similar to GPs. Both of them are composed of chondrocytes and cartilage matrix, they are divided into different zones according to the differentiational state of chondrocytes. Their main components of ECM are Col II, Col X, and glycosaminoglycans(GAGs). However, there are also some differences between GPs and articular cartilage. Firstly, from the perspective of developmental biology, the GP is most affected by age. In short bones, like phalanges, the GP will close early, while in long bones, it will close later (Lui et al., 2018). When people reach adulthood, all GPs will be replaced by bone plates. In traditional assumptions, it is eventually programmed to cease at the age of 14 in girls and 16 in boys (Little and Milewski, 2016). However, articular cartilage will maintain function and degenerate until people get old. Secondly, in terms of tissue structure, GPs have three distinct zones while articular cartilage is divided into four zones: the superficial zone, the middle zone, the deep zone, and the calcified zone (Qiao et al., 2021). Meanwhile, the arrangement of chondrocytes in GPs are more regular in a columnar structure. Unlike the junction between bone and articular cartilage, GPs have two chondro-osseous junctions because of their anatomical locations (Kazemi and Williams, 2020). In addition, the ECM between GPs and articular cartilage are different as well. As Diaz-Payno et al. (2020) reported, GP specific ECM could be used to promote osteogenesis of BMSCs while articular cartilage derived ECM was potent for chondrogenesis. The spatial distribution of growth factors was also different. In the GP, expression of BMP2 and BMP6 are increased from resting zone to hypertrophic zone. But in articular cartilage, they are decreased from superficial to calcified zones (Garrison et al., 2017). Finally, the functions of GPs and articular cartilage are totally different. The GP is mainly responsible for longitudinal growth, which is vulnerable and cannot bear much compression, especially for the hypertrophic zone (Xie et al., 2020). Articular cartilage is located in joints, which plays the role of lubrication and buffering (Matsuzaki et al., 2018).

### Pathological Characteristics of the GP Damage

#### Classification of GP Injuries

The Salter-Harris (SH) Classification System is most commonly used classification in clinical use that classifies GP injuries into five patterns (Figure 1B). About 5% of GP injuries are SH type I, where the injuries affect the whole GP and produces fragmentation. SH type II is the most common type of injury and accounts for 75% of observed injuries, whereby the injuries not only occur transversely across the GP, but also obliquely penetrate the metaphysis. In SH type III, the injuries cross the GP and obliquely penetrate the epiphysis, although this type only accounts for 10% of injuries. In SH type IV, the injuries occur longitudinally through the GP from the articular surface to the metaphysis, this type occurs in 10% of all GP injuries. SH type V, the compressional type, is the least common injury, but is the most likely type to result in bone bridge formation (Chung and Xian, 2014; Sferopoulos, 2014). Among these patterns, the more superficial injuries (SH type III, IV, and V) that destroy both the GP and its blood supply often lead to growth arrest and bone bridge formation, the deeper injuries (SH type I and II) which do not disturb the blood supply usually achieve a better prognosis (Yukata et al., 2018; Watanabe et al., 2019).

#### Phases of GP Injuries

**FIGURE 2:**
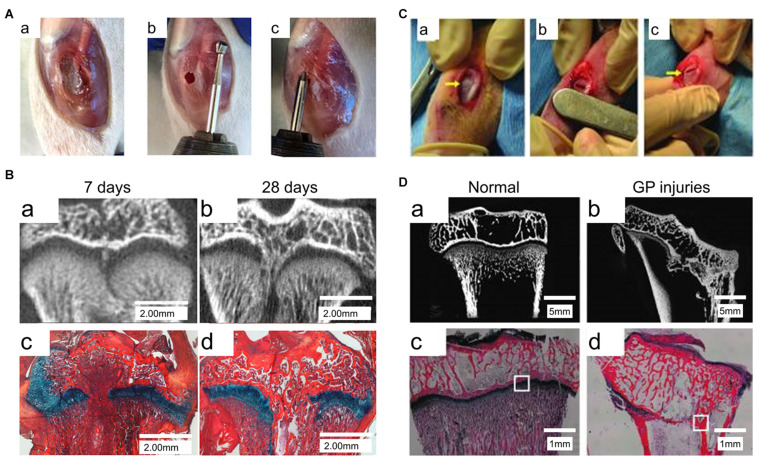
Animal models of GP injuries. **(A)** The rat model of proximal tibia GP injuries. **(B)** Micro-CT and Alcian Blue Hematoxylin staining with Orange G/Eosin counterstain (ABH stain) of the GP injuries at 7 days and 28 days post-injury (Erickson et al., 2017). **(C)** The rabbit model of proximal tibia GP injuries. **(D)** X-ray microscopy and ABH stain of the GP injuries (Yu et al., 2019).

In previous studies, numerous animal models have been used to elucidate the pathophysiology involved in bone repair in GP injuries (Figure 2). These animal models included immature mice (Erickson et al., 2017), the miniature pig (Ding et al., 2018), and sheep models (Knapik et al., 2018). These studies identified four phases of injury responses leading to bone repair, namely, the inflammatory phase, the fibrogenic phase, the osteogenic phase, and the remodeling phase (Zhou et al., 2004, 2006).

**FIGURE 3:**
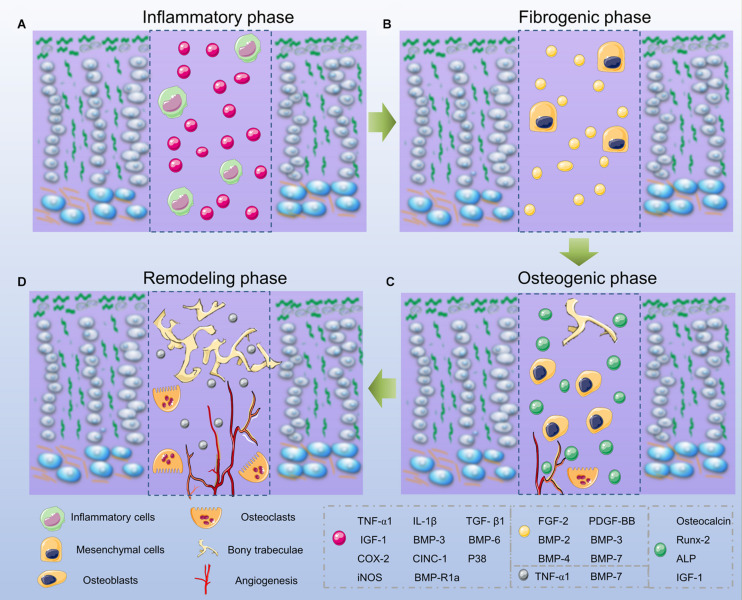
Phases of bone bridge formation. **(A)** The inflammatory phase. **(B)** The fibrogenic phase. **(C)** The osteogenic phase. **(D)** The remodeling phase.

As for common bone fracture and soft tissue injuries, the first phase after GP injuries is the inflammatory phase, and involves an initial influx of inflammatory cells including neutrophils, macrophages/monocytes, and lymphocytes (Figure 3A). Consistent with this infiltration, the rat neutrophil chemokine CINC-1, equivalent to human interleukin (IL)-8, peaks on day 1 and decreases to basal levels on day 4 (Chung et al., 2006, 2011; Chung and Xian, 2014). In order to confirm the importance of the neutrophil-mediated inflammatory responses in bone repair, Chung et al. (2006) utilized a neutrophil-neutralizing antiserum in rats, and their results showed an increase of osteogenesis-related genes like osteocalcin and core binding factor α-1, a decrease of chondrogenesis-related genes like sex determining region Y box-9 (Sox-9) and Col II, which suggested the inflammatory phase was vital for downstream bone repair events. In addition, TNF-α and IL-1α also increase significantly on day 1 (Wang et al., 2017). Using a TNF-α antagonist, Zhou et al. (2006) found a clear delay of mesenchymal infiltration, which means TNF-α is required for the migration and proliferation of mesenchymal cells. Other studies also highlighted that TNF-α is a critical factor for healing and tissue repair (Birkl et al., 2019). Overall, the inflammatory phase plays a significant role in the repair of GP injuries as it modulates the cascade downstream of the healing responses.

After the initial inflammatory response, the fibrogenic phase appears on days 3–7 after the GP injuries and involves the fibrous vinmentin-immunopositive mesenchymal cells gathering at the injured site. These cells may contain mesenchymal stem cells (MSCs), osteoprogenitor, and chondroprogenitor cells, which are confirmed to be vital for the fracture repair process (Macsai et al., 2011; Neumayer et al., 2017; Figure 3B). In the fibrogenic phase, growth factors, such as FGF-2 and platelet derived growth factor (PDGF) play an important role (Zhou et al., 2004; Shaw et al., 2018). FGF-2 in particular acts by stimulating osteoprogenitor and mesenchymal cell proliferation, migration, alkaline phosphatase (ALP) activity, as well as inhibiting chondrocyte differentiation (Nasrabadi et al., 2018). During wound healing, PDGF functions to enhance cell migration, proliferation, and angiogenesis. During repair of GP injuries, PDGF is essential for the proliferation and migration of fibroblasts and osteoblasts (Zhou et al., 2004; Chung et al., 2009, 2011).

Around day 7, the subsequent osteogenic phase occurs with the appearance of trabecular bone. The Runt-related transcription factor 2 (Runx-2) and ALP-positive stained cells are observed. Additional bone matrix proteins including osteocalcin (OCN) are produced (Xian et al., 2004). Similarly, Col I production is observed by the presence of regenerated bone tissue at the injury site. During this phase, the bone bridge begins to form (Chung and Xian, 2014; Figure 3C).

The remodeling phase can be observed from day 14 onwards, since there are more mesenchymal cells and osteoclasts present in the bone trabeculae (Chung et al., 2009). During this phase, chondrogenesis-related genes like Sox-9 and Col II are expressed at a low level, while the osteogenesis-related genes like OCN are highly expressed (Zhou et al., 2004). In addition, growth factors, such as TNF-α, IGF-1, and BMP-7, increase as well, and they promote differentiation and recruitment of the osteoclasts, thus promoting bone remodeling (Fischer et al., 2018; Kim et al., 2018; Figure 3D).

#### Responses to GP Injuries in Different Anatomical Locations

After the GP injury, chondrocyte columns become disorganized at the injury site (Hajdu et al., 2011). Throughout the whole section, the thickness of the injured area was always higher than non-injured area. As more hypertrophic chondrocytes occurred after the fibrogenic phase, the height of resting zone and hypertrophic zone increased while proliferative zone height relatively reduced. Immunohistochemistry of Col X staining also reported that Col X extends throughout the entire injury site (Drenkard et al., 2017). Furthermore, the GP injury also affects the adjacent non-injured region because injuries destroyed the controlled endochondral ossification process. For example, Macsai et al. (2011) detected bone bridge formation at the uninjured area of GPs on day 60 in rats. Further analysis revealed there was a decrease in expression of chondrogenic factors including Sox-9, transforming growth factor-β1 (TGF-β1), and IGF-1, and an increase of apoptotic factors like caspase-3 in the adjacent non-injured area (Musumeci et al., 2013). Studies have demonstrated there are different responses in varied locations after GP injuries, but the detailed evidence is limited. It requires more studies to elucidate how the three distinct zones response in varied phases and how these contributions change the outcomes after GP injuries.

## Cartilage Tissue Engineering for GP Injuries

The engineering of cartilage tissue is a comprehensive approach that utilizes various cell types and growth factors, including bone marrow mesenchymal stem cells (BMSCs), chondrocytes, and TGF-β, IGF-1, and FGF-2 (Chen et al., 2020; Wei et al., 2020), as well as different scaffolds constructed with natural or synthetic materials (Abdollahiyan et al., 2020). In this section, we will discuss the progress of cartilage tissue engineering in the treatment of GP injuries.

### Seed Cells

Because of the limited microenvironment for cartilage regeneration, seed cells are widely used to fill defects following bone bridge resections. By promoting cell proliferation and ECM excretion, the seed cells will restore the cartilage tissue of the GP. Extensively used seed cells, such as MSCs and chondrocytes in GP repair are discussed below.

#### Mesenchymal Stem Cells

MSCs have been widely used in cartilage tissue engineering due to their capability for self-renewal and potential for multiple differentiation. MSCs can secrete diverse growth factors and differentiate into various cellular types, such as osteocytes, chondrocytes, and adipocytes, which play an important role in cell-based therapies (Isobe et al., 2016; Gultekin et al., 2020). As mentioned earlier, during the fibrogenic phase, there is an influx of MSCs in the injured site, indicating that MSCs are vital in the repair process of GP injuries (Zhou et al., 2004).

In previous studies, MSCs from multifarious sources were utilized in the treatment of GP injuries and achieved excellent results (Sananta et al., 2020). Li et al. (2017) fabricated an oriented ECM scaffold incorporating BMSCs to cure the injured GPs in rabbits, and results showed that compared to ECM scaffolds alone, ECM scaffolds with BMSCs prevented the bone bridge formation, reduced the length discrepancy and consequently the angular deformity. To further examine the role of periosteum MSCs, Chen et al. (2003) transferred the periosteum together with harvested MSCs embedded agarose to the site of GP defects, the transferred group receiving agarose alone showed poor results, while the angular deformity and growth arrest were corrected in the MSCs embedded group. In another study, Ando et al. produced a 6-week-old rabbit growth arrest model by disrupting the medial half of the proximal tibias, in order to test the effect of synovial derived MSCs, a scaffold-free construct was used, and results showed that the MSCs proliferated and differentiated into cells similar to chondrocytes, suggesting that the MSC-based therapy could be an effective method for curing GP injuries (Yoshida et al., 2012).

In addition to MSCs derived from bone marrow, periosteum, and synovium, MSCs can also be obtained from adipose tissue, umbilical cord, placenta, and skeletal muscle (Uder et al., 2018). Previous studies have demonstrated that MSCs derived from various compartments possess different regenerative potentials (Kaviani et al., 2019). Therefore, it is necessary to define the most practical way to promote the GP repair for clinical applications among all the available sources. Therefore, Isobe et al. (2016) examined the multipotentiality of MSCs derived from adult dental pulp, synovial fluid, exfoliated deciduous teeth, and bone marrow, and concluded that bone marrow- and synovial fluid-derived MSCs were most suitable for osteogenesis while synovial fluid-derived cells produced the highest levels of chondrogenesis. Similarly, Sakaguchi et al. (2005) also compared the properties of MSCs derived from bone marrow, periosteum, adipose tissue, and skeletal muscle, and also verified that MSC features differed significantly according to their sources, and MSCs isolated from synovium were superior in both osteogenesis and chondrogenesis. Although synovium-derived MSCs seemed to have more potential for GP repair, they are more difficult to obtain and purify, which limits their application. Meanwhile, BMSCs can be isolated easily and are expanded efficiently (Yee et al., 2018). Furthermore, it has been demonstrated that BMSCs can stimulate angiogenesis, suppress the immunoreaction, and inhibit fiber tissue formation (An et al., 2018). Altogether, the evidence suggests that BMSCs maybe more suitable as seed cells in the treatment of GP injuries (Planka et al., 2009).

As for MSCs, another problem that has been addressed is whether autogenous MSCs are superior to allogeneic MSCs. In one clinical study, patients with Hurler syndrome (MPS-IH) infused allogeneic BMSCs for treatment of patients with metachromatic leukodystrophy (MLD). The results indicated an improvement in bone mineral density and nerve function among all patients, indicating that allogeneic MSCs could survive and function in host tissue (Koc et al., 2002). Furthermore, Planka et al. (2008) transplanted autogenous and allogeneic MSCs in rabbits with distal femoral GP injuries, and found there was no significant difference either in femur length discrepancy or in angular deformity between these two procedures. It seems there is a high tolerance of allogeneic MSCs in host immune rejection. Studies have elucidated that allogeneic MSCs escape from the host immune response by altering cytokine secretion, and thus modulate immune cells including dendritic cells, natural killer cells, and effector T cells (Bocelli-Tyndall et al., 2007; Cequier et al., 2019). Therefore, the effects of allogeneic MSCs are equal to those of autogenous MSCs.

#### Chondrocytes

Since the most abundant cell type in the GP is chondrocytes, it is quite rational to implant chondrocytes in cartilage tissue engineered scaffolds for treatment of GP injuries (Lee et al., 2016; Tomaszewski et al., 2016). Chondrocytes can also be obtained from different compartments in autograft or allograft. In an autologous chondrocyte experiment, Tomaszewski et al. (2014) resected the medial part of the proximal tibia GP in rabbits and then implanted it in the GP defects. Histological and radiological results demonstrated that implanting of autologous chondrocytes significantly prevented the bone bridge formation and growth arrest. In another study, Jin et al. (2006) investigated autologous chondrocytes obtained from the iliac crest, seeded on the demineralized bone matrix (DBM) scaffold for the treatment of rabbit GP injuries. This type of autologous tissue engineered scaffold not only prevented the angular deformity and bone formation, but also built the columnar structure at the injured site. Although the autologous chondrocytes can avoid the immune rejection and show good results both *in vitro* and *in vivo*, they are limited in number and may cause additional damage (Boopalan et al., 2019). From this standpoint, allogeneic chondrocytes may represent a better alternative if they present good results in future studies. Li et al. (2013) used allogeneic chondrocytes harvested from distal femoral GPs, microencapsulated by semipermeable membranes, and transplant the preparations in a GP injury model. Sixteen weeks later, the chondrocytes-treated group showed less length discrepancy and angular deformity than other groups, the histological results also exhibited columnar arrangement formed by neogenetic chondrocytes at the injured site, which indicated that the allogeneic chondrocytes could prevent bone formation to the same extent as autologous chondrocytes. Since there is no significant difference between allogeneic and autologous chondrocytes, it is tempting to speculate how allogeneic chondrocytes escape from the immune response. Several studies have proposed that the avascular nature in cartilage and the surrounding ECM may provide a protective immune barrier for embedded chondrocytes (Zhao et al., 2019).

Allogeneic and autologous chondrocytes may have a similar treatment effect, but when determining the body sites for chondrocyte derivations, it is difficult to define the most suitable site. Although Jin et al. (2006) indicated that chondrocytes derived from the iliac crest GP had more advantages than chondrocytes from joint cartilage as the former still had the potential for proliferation and differentiation (Jin et al., 2006). More studies are needed to elucidate the different effects between chondrocytes isolated from various sources.

As for proliferative and differentiation abilities, *in vitro* studies have shown that three dimensional (3D) cultures can retain the chondrogenic potential better than monolayer cultures (Chow et al., 2011). Parreno et al. (2017) demonstrated that the monolayer culture of chondrocytes could alter their phenotype and produce more Col I secretion and less Col II secretion, which indicated the primitive feature of chondrocytes was lost. Meanwhile, several studies have demonstrated that 3D culture of chondrocytes can promote cellular proliferation without changing phenotypes. For example, rabbit articular chondrocytes cultured on poly(ethylene glycol)/poly(ε-caprolactone) (PEG/PCL) hydrogel led to the up-regulated expression of chondrogenic genes such as Sox-9, aggrecan, and Col II in 2 weeks, and increased proteoglycans and Col II accumulation after 4 weeks (Chang et al., 2018). Therefore, in order to retain the phenotype of implanted chondrocytes, a favorable strategy is to incubate them in a 3D culture environment before seeding.

### Growth Factors

Cartilage-stimulating growth factors are bioactive peptides that bind to specific receptors and trigger a series of cell activities including cell migration, proliferation, and differentiation (Chen et al., 2020). In order to ameliorate the microenvironment for cartilage tissue formation, it is necessary to use chondrogenic factors such as IGF-1, FGF-2, and TGF-β1, to stimulate the chondrogenic differentiation of chondrocyte-related progenitor cells.

#### TGF-β

TGF-β is produced in an inactive form and is activated via signaling pathways, it plays an important role during the chondrogenesis of MSCs (Chen et al., 2018). *In vivo*, TGF-β has two forms, and mostly accumulates by binding to the ECM, while the other form is a soluble free form, which is present in only small amounts, but plays a predominant role. Previous studies have demonstrated that TGF-β functions differently and has opposite effects on GP through TGF-β/Smad2/3 or BMP/Smad1/5/8 signals. The TGF-β/Smad2/3 signaling pathway stimulates chondrogenesis and ECM synthesis while the BMP/Smad1/5/8 signaling pathway inhibits chondrogenesis and promotes osteogenesis (Thielen et al., 2019). As a short-lived cytokine, TGF-β is only active for a few minutes in response to GP inflammation or ECM damage (Liu W. et al., 2020).

Since TGF-β is a critical factor in cartilaginous differentiation, cartilage tissue engineered scaffolds have used TGF-β in the treatment of GP repair. An *in vitro* study indicated that MSCs induced by TGF-β1 presented significantly higher levels of aggrecan, Col II, and Sox-9 in a high-density monolayer culture (Coleman et al., 2013). In an ovine animal model, McCarty et al. (2010) utilized a gelfoam scaffold containing autologous BMSCs and TGF-β1, implanted in the proximal ovine tibial GP defect, and results showed that the scaffold containing TGF-β1 inhibited bone bridge formation.

Two strategies have been described for using TGF-β to stimulate MSC differentiation or proliferation: one involves the secretion of TGF-β by chondrocytes through co-culture of MSCs and chondrocytes, the other involves the addition of exogenous TGF-β. Chen et al. (2018) used a mathematical model to compare these two distinct strategies and proposed a hybrid strategy. The authors reported that in cocultures of chondrocytes and MSCs, a critical value of chondrocyte density was to be achieved before the complete differentiation of MSCs could be induced. For the *in vitro* environment, the critical density was between 5 and 25%. With regard to the exogenous administration of TGF-β, there were two critical values, a_*crit1*_ and a_*crit2*_: below the concentration of a_*crit1*_, no cells were produced, and above the initial concentration of a_*crit2*_, all MSCs would be driven to differentiation. The value of a_*crit2*_ was slightly lower than 10 ng/mL. Moreover, by combing these two strategies, fewer chondrocytes were required and less exogenous TGF-β was needed to induce MSCs differentiation, and a lower concentration of a_*crit2*_ was needed, requiring about 10% chondrocytes co-culture (Chen et al., 2018). Similarly, Dahlin et al. (2014) also observed that co-culture of articular chondrocytes and MSCs required less TGF-β3 to achieve an equivalent chondrogenesis level compared to MSCs cultured alone. In conclusion, it is more effective to use chondrocyte co-cultures and less exogenous TGF-β to stimulate MSCs differentiation or proliferation.

#### IGF-1

Being vital in cartilage homeostasis and repair, IGF-1 is an anabolic growth factor which has been extensively studied (Lo et al., 2020). Previous studies have confirmed that IGF-1 can not only stimulate chondrocytes to synthesis matrix proteins like Col II and proteoglycan, but it can also inhibit chondrocytes degradation and apoptosis during cartilage damage by blocking the function of IL-1 or TNF-α (Mahran et al., 2019). In a clinical trial, IGF-1 was used to treat short stature children for 1 year. No adverse events were reported, which indicated that IGF-1 may have potential for clinical application (Midyett et al., 2010).

**FIGURE 4:**
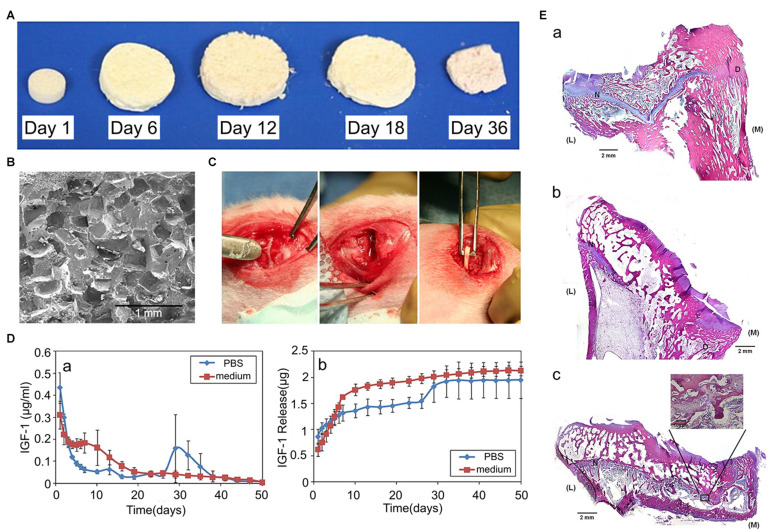
Effects of IGF-1 loaded scaffolds in GP injuries. **(A)** Morphology of the PLGA scaffold and its degradation in the cell culture medium. **(B)** The transverse plane of PLGA scaffolds observed using a scanning electron microscope (SEM). **(C)** The proximal tibial GP defects of rabbits. **(D)** The biphasic pattern of IGF-1 release. **(E)** Hematoxylin and eosin (H&E) staining staining of the GP repair. (a) control group without implantation, (b) the blank scaffold group, (c) the IGF-1 loaded scaffold group (reproduced with permission from Sundararaj et al., 2015).

Over the past 20 years, several studies have been conducted to investigate the effects of IGF-1 on loaded scaffolds in GP regeneration. In an *in vitro* study, Mullen et al. (2015) utilized a porous collagen-glycosaminoglycan scaffold containing chondrocytes and different concentrations of IGF-1, and testing the amount of proteoglycan and Col II products. The results showed that the most suitable IGF-1 loading concentration was 50 μg/mL, and IGF-1 loaded groups synthesized more ECM than the empty group (Mullen et al., 2015). An *in vivo* study also indicated that a collagen sponge impregnated with exogenous IGF-1 induced higher chondrocytes influx and ECM production in immature cartilage defects, which means that IGF-1 is beneficial to cartilage repair (Tuncel et al., 2005). In another study, porous PLGA scaffolds loaded with IGF-1 were used in the treatment of a rabbit model with proximal tibial GP defects, after implantation in the GP defects, regenerated cartilage was observed in the IGF-1 releasing group, while there was only bone formation in the empty group and in the scaffolds alone group (Figure 4), all the results indicated that IGF-1 was suitable for GP regeneration (Sundararaj et al., 2015). As for the biphasic pattern of IGF-1 release in PLGA scaffolds, initially, due to the rapid surface diffusion, a burst in IGF-1 release is observed within 24–48 h, this burst can be therapeutic for it initiates early MSCs differentiation, proliferation, and ECM deposition. Afterward, IGF-1 is released to a much lower degree with erosion of the scaffold matrix, thus maintaining a certain concentration of IGF-1 in the injured site (Giteau et al., 2008; Mullen et al., 2015; Sundararaj et al., 2015).

#### FGF-2

The family of the FGF has been demonstrated to be critical for a wide range of cell types regarding differentiation, proliferation, migration, and growth. Among the FGF family members, FGF-2, FGF-8, and FGF-18 have recently been proposed to be the most important contributing factors in cartilage modulation (Lee et al., 2018). As for GP-related MSCs differentiation and proliferation, FGF-2 has been extensively investigated. Interestingly, previous studies have shown contradictory effects for FGF-2 in the expansion and differentiation phases (Jin et al., 2020). During MSCs expansion, FGF-2 enhances the proliferation potential and retards the differentiation process by regulating the expression of FGF receptor 1 (Yang et al., 2008). Moreover, by upregulating FGF receptor 3, it also promotes matrix deposition (Furusho et al., 2020). During the differentiation phase, the FGF receptor 1 is poorly expressed, and thus inhibits differentiation and matrix deposition (Han et al., 2020).

FGF-2 is effective when applied to cartilage tissue engineered scaffolds for GP repair which mainly exist in the resting and proliferating zones (Krejci et al., 2007). An *in vitro* study indicated that MSCs expanded and maintained high viability of FGF-2 levels, but showed minimal matrix deposition (Coleman et al., 2013). Other MSCs culture experiments also demonstrated that MSCs express FGF receptors, and FGF-2 treatment increased the mitogenic ability of MSCs, thus promoting their proliferation rate during expansion (Xu et al., 2017). In the application of engineered cartilage tissue, FGF-2 was usually used with other growth factors. In an experiment of rat BMSCs culture, Coleman et al. (2007) demonstrated that BMSCs produced greater amounts of sulfated glycosaminoglycans in the presence of FGF-2 and TGF-β1 than with TGF-β1 treated alone, which indicates that FGF-2 plays a role in GP regeneration.

#### Other Growth Factors

In addition to the growth factors described above, (i.e., TGF-β, IGF-1, and FGF-2) there are many other growth factors that have also have been validated to be associated with GP repair.

Using a rat tibial GP injury model, Chung et al. (2014) found that treatment with anti-VEGF antibody inhibited the activity of VEGF, which decreased bone formation, OCN, and Runx-2 expression, indicating that VEGF-promoted angiogenesis plays an important role in undesired bone repair. Another study found that neurotrophin-3 enhanced osteogenesis and angiogenesis by upregulating BMP-2 and VEGF in bone formation, indicating that neurotrophin-3 may be a potential target to inhibit bone repair in GP injuries (Su et al., 2016). Similar to VEGF and BMP-2, BMP-6, and BMP receptor-1a were also shown to contribute to bone formation (Fischerauer et al., 2013). However, Zhou et al. (2006) demonstrated that TNF-α could inhibit bone formation at the injured GP site in rats by stimulating p38 MAP kinase activity. In conclusion, these growth factors are positively or negatively associated with unwanted bone formation during GP repair, they are potential targets for curing GP injuries. However, the therapeutic application of these growth factors in cartilage tissue engineering have not been fully explored. Further studies are required to elucidate their roles for GP regeneration.

### Scaffolds

Since the GP is a functional area responsible for longitudinal growth and withstands weight-bearing between the metaphysis and diaphysis, it is important for cartilage scaffolds to exhibit good biocompatibility, biodegradability, suitable porosity, as well as appropriate mechanical properties. Currently, a wide range of biomaterials have been used for GP repair, including natural and synthetic materials.

#### Fabrication Methods

In cartilage tissue engineering, various preparation methods have been applied while fabricating scaffolds. These methods include the freeze-drying technique, the salt leaching technique, 3D printing technique, the gas foaming technique, electrospinning technique, crosslinking methods for hydrogel scaffolds preparation and so on. In this section, we will present the most commonly used technique in GP scaffold fabrication.

##### The Freeze-Drying Technique

The freeze-drying technique has been commonly used for fabricating porous scaffolds in previous investigations. It contains three major phases: first, the material solutions are infused into a cylindrical mold, which is then allowed to freeze in a freezer at −20°C for 1 or 2 h. Then, the mold is transferred into a freeze dryer to allow lyophilization under vacuum for approximately 48 h. Finally, the scaffold is then dried and stored in a refrigerator at 4°C (Suphasiriroj et al., 2009; Zheng et al., 2011). Scaffolds manufactured using this method have significant advantages, generating pore sizes of around 100 μm and porosity of more than 90%, and with some improvements, it can also produce structured scaffolds, which not only improve mechanical properties, but also exhibit a biomimetic columnar structure to stimulate chondrocytes proliferation in GPs (Mohammady et al., 2020). However, freeze-drying is a complex and time consuming process with harsh environmental requirements and the need for expensive equipment limit its applications. Nonetheless it is still challenging to find new techniques with simpler procedure and cheaper cost.

##### The Salt Leaching Technique

The salt leaching technique is mainly used to fabricate scaffolds using materials in the form of microspheres. The procedure can be illustrated briefly as follows: initially the microspheres are mixed with salt particles, mostly NaCl. Afterward, the mixture is placed in a cast (according to the diameter of scaffolds) and is consolidated by compressing at a load of 2.5 tons (tons increase with diameters) for a few minutes. Then, the disc is heated near the melting point (Tg) of the material for about 48 h in order to fuse the microspheres. After that, the salt is leached in deionized water overnight to create porous scaffolds. Finally, the porous scaffolds are lyophilized in a vacuum (Das et al., 2018). Following these steps, the scaffolds are ready to use. The pore size achieved by this method is between 1 and 700 μm, the porosity is around 60% (Ravi et al., 2012). Although scaffolds made in this way have good mechanical properties, the procedure is also time consuming and complex, the available materials are limited and the pore sizes are randomly distributed. Therefore, it is necessary to find another technique which can control the pore size in a more accurate way.

##### 3D Printing Technique

Application of 3D printing, or additive manufacturing, also known as solid-freeform technology or rapid prototyping, is a promising technology developed since the mid-1980s, and has been applied in various fields including construction, automation, and aerospace. The additive manufacturing techniques include stereolithography (SLA), fused deposition modeling (FDM), selective laser sintering (SLS), inkjet bioprinting, and extrusion bioprinting (Reeser and Doiron, 2019). Compared to traditional techniques used to make scaffolds for the treatment of GP injuries, 3D printing has the following advantages. (1) Few equipment and technical requirements: to complete the scaffold, all that is needed are prepared materials, a 3D printer, and a computer-aided design (CAD) software (Mikolajczyk et al., 2019). There is no need to modify temperature or time, the 3D printer automatically adjusts these parameters (Siller et al., 2019). (2) Lower costs and time saving: with minimal use of materials and printing requires only a few hours or even a few minutes, thus demanding less labor and material resources, the 3D printing technique can maximally cut the cost (Ballard et al., 2020). (3) A wide range of available materials: unlike conventional fabricating techniques, 3D printing uses a variety of materials, including metals, alloys, polymers, and bioceramics (Tardajos et al., 2018). (4) Precise individual customization: through a CAD software monitor, the pore size and porosity are made highly consistent with expectations, consequently, it is possible to fabricate gradient scaffolds with accurately designed pore sizes (Wo et al., 2020).

##### Hydrogel Scaffolds Preparation Techniques

Nowadays, hydrogels are immensely used in cartilage tissue engineering because of their excellent biocompatible properties. They have three dimensional networks which are formed from crosslinked polymer. They provide desired structure by absorbing water, which mimic the natural ECM of cartilage (Choi et al., 2020b). Various hydrogel scaffolds have been used in cartilage tissue engineering, their composition include natural materials (e.g., alginate, chitosan, Col and gelatin), synthetic materials [e.g., polyether, poly(vinyl pyrrolidone), and poly(acrylate acid)] and composite materials.

As for preparation of hydrogels, they are crosslinked by physical or chemical crosslinking methods. Physical crosslinking is a way to produce network of polymer chains by physical treatments like heating, cooling, freeze-drying, or ultrasonication. In this way, the polymer networks are connected by reversible bonds such as ionic interaction, hydrogen bonds, or crystallization (Zhang et al., 2021). The advantage of physical crosslinking methods is that each component does not produce chemical reaction during crosslinking. It avoids the production of new substances which may be toxic. Its deficiency is obvious as well. Hydrogels made in this way are deficient in mechanical strength and thermal stability (Wang Y. X. et al., 2020). Materials suitable for physical crosslinking are chitosan, Col I, alginates, and polyvinyl alcohol. Chemical crosslinking is another gelation method which creates covalent linkage among polymer chains by using proper crosslinking reagents (Kong et al., 2020). These crosslinking reagents include glutaric dialdehyde, tannic acid, genipin and so on. Because of the strong connections, chemical crosslinking not only enhances mechanical strengths of hydrogels, but also improves the resistance to degeneration. Moreover, the biomechanical strengths can be adjusted by altering the type or concentration of reagents (Fathi-Achachelouei et al., 2020). The main disadvantage of chemical crosslinking method is the potential usage of cytotoxic reagents, catalysts, or initiators (Oryan et al., 2018).

For preparation of hydrogel scaffolds, various techniques have been used, such as bioprinting technique, microfluidic technique, photolithography technique and so on. Among these techniques, bioprinting technique is a 3D printing technique using hydrogels as a bioink (Antich et al., 2020). Besides bioprinting, microfluidic technology is another efficient method to fabricate hydrogel scaffolds with precision. Through droplet production methods like dielectrophoresis and electrowetting on dielectric, microfluidic systems can produce microparticles in a highly monodispersed pattern and manipulate these nanoliter of liquids through microchannels. Because of the production of tiny droplets, this technique is a powerful tool for preparing scaffolds with complex 3D structure, as well as hydrogel scaffolds with mechanical or chemical gradients (Moreira et al., 2021). The photolithography technique is mainly used in hydrogel that crosslinked via ultraviolet light. After fabricate hydrogel in a plane pattern, the light will transmit through the hydrogels in designed pathway. The mechanical strength of scaffolds can be altered by changing the intensity or irradiation time of ultraviolet light (Vedadghavami et al., 2017).

#### Materials

##### Natural Materials

Thanks to the superior biocompatibility and suitable biodegradability, natural materials like ECM, alginate, agarose, and chitosan are appropriate to initiate chondrocyte regeneration and cartilaginous ECM secretion (Choi et al., 2020a).

###### ECM Derived From GPs.

As a natural material derived from GP, ECM is an alternative matrix used to make scaffolds for treatment of GP injuries, since it is not only composed of cartilage matrix such as GAGs and Col II which can best imitate the microenvironment for chondrocyte regeneration (Cunniffe et al., 2019), but it is also known to contain various growth factors that modulate angiogenesis, cell migration, differentiation, proliferation, and the immune response (Horton et al., 2020).

**FIGURE 5:**
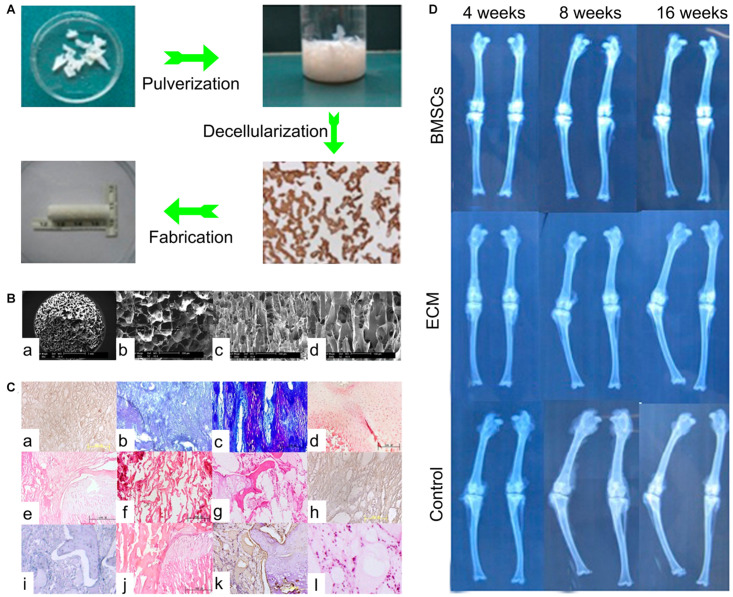
The ECM scaffold in treatment of GP injuries. **(A)** The process of ECM scaffold fabrication. **(B)** SEM micrograph of ECM scaffolds. **(C)** The histological results of ECM based scaffolds. (a–d) In the ECM + BMSCs group, from 4 to 16 weeks, neogenetic chondrocytes increased gradually and were arranged in a columnar structure. (e–h) In the ECM alone group, from 4 to 16 weeks, fibrous tissue and bone tissue gradually come into being. (i–l) In the control group, from 4 to 16 weeks, fibrous tissue and bone tissue covered the defects early. **(D)** The radiological results of three groups [Reproduced with permission from Li et al. (2017)].

Previous studies have shown that GP-derived ECM containing diverse growth factors, such as VEGF and IGF-β1, not only supported vascularization, but also enhanced the regeneration of BMSCs (Cunniffe et al., 2017). These results suggested that this type of ECM was a multipotential substrate and its function would change based on the transplantation site. For treatment of GP injuries, Li et al. (2017) collected the ECM from GPs and constructed the structured ECM scaffolds loaded with BMSCs. Sixteen weeks after transplantation into the tibial GP defects, the histological results showed regeneration of new chondrocytes, and the radiographic results showed reduced length and angular deformities (Figure 5), indicating this GP generated a structured ECM scaffold with the potential for GP repair (Li et al., 2017). Although GP-derived ECM was biocompatible with the porous structure and proved superior to artificial polymer materials, the restricted accessibility limits its application (Lee S. et al., 2019). For clinical usage, it is necessary to find materials that are more accessible, inexpensive, biocompatible, and biodegradable.

###### Alginate.

With ideal biocompatibility and low toxicity, alginate is one of the most extensively used materials for hydrogel-based cartilage tissue engineering (Sturtivant and Callanan, 2020). Since alginate hydrogels are able to provide a 3D environment with a wide range of pore sizes, scaffolds made by alginate facilitate MSCs distribution and provide efficient nutrient transport (Farokhi et al., 2020). Furthermore, it can also be used to deliver growth factors with adjustable release rates by changing the molecular weight (Jiao et al., 2019). In order to enhance the chondrogenesis of MSCs in alginate, several studies have successfully delivered growth factor or genes to local MSCs, thus directs the fate of MSCs and increases sGAG and Col II production (Davis et al., 2018; Khatab et al., 2020). As for GP cartilage regeneration, using an *in vitro* model of the GP chondrocytes seeded on alginate hydrogel scaffolds with exogenous factors also resulted in high viability, low level hypertrophy, and cartilage matrix deposition (Coleman et al., 2007; Erickson et al., 2018). In a GP injuries model, alginate-polylysinealginate semipermeable membranes were used for chondrocyte encapsulation. After 16 weeks of implantation into defects, the radiological results showed less angular deformity and length discrepancies, indicating this alginate material was suitable for GP reconstruction (Li et al., 2013). However, alginate hydrogel is negatively charged, which results in a dissimilar environment for encapsulated cells (Freeman and Kelly, 2017). Nonetheless, alginate hydrogel is still a suitable material for constructing scaffolds for GP regeneration.

###### Agarose.

Agarose is another popular material for cartilage tissue engineering. As a natural polysaccharide polymer, agarose is composed of repetitions of D-galactose and 3, 6-anhydro-L-galactopyranose (Choi et al., 2020a; Salati et al., 2020). Similar to alginate, it is commonly used for hydrogel due to its excellent biodegradability and biocompatibility as well (Bonhome-Espinosa et al., 2020). Moreover, agarose has a similar structure to ECM, and possess great capacity of water absorbing, both features make it particularly suitable for cell adhesion, cell growth, differentiation, and nutrient permeation (Grolman et al., 2019). When applied to treatment of GP defects with MSCs, both *in vitro* and *in vivo* studies presented chondrogenesis and correction of limb deformity, indicating agarose scaffolds could support cell growth and delivery growth factors (Chen et al., 2003; Coleman et al., 2013). Most importantly, its thermal reversible gelation behavior and internal networks allow it to composite with other polymers, which makes it possible to fabricate scaffolds with higher strength (Lee Y. et al., 2019).

###### Chitosan.

Chitosan, made of β(1–4) glycosidic bonds and D-glucosamine residues, is a natural polymer found in the exoskeleton of crustaceans (Ribeiro et al., 2020). It contains different amounts of N-acetyl-D-glucosamine (NAG) groups. When the chitosan has more than 50% NAG, it is called chitin, and when it has more than 50% N-glucosamine, it is called chitosan (Saravanan et al., 2016). With the characteristics of excellent mechanical stability, biocompatibility, biodegradability, and a hydrophilic surface, chitosan is thought to be a suitable material to fabricate porous scaffolds (Ishikawa et al., 2020). When applied to cartilage tissue engineering, it has shown to promote hyaluronic acid synthesis, which benefits cartilage regeneration (Kashi et al., 2018). To restore the damaged GP, the chitosan scaffold alone showed poor results, but when combined with a large concentration of MSCs, it resulted in less angular deformity in rabbits (Azarpira et al., 2015). Therefore, MSCs based chitosan scaffolds may be a good combination in treatment of GP injuries (Erickson et al., 2020).

##### Synthetic Materials

Synthetic materials, such as PLGA, PLA, and PCL, are widely used to make scaffolds in cartilage tissue engineering, they have tunable properties in terms of mechanics and degradation rates can be artificially regulated by changing the degree of polymerization (Uz et al., 2019). Compared to natural materials, they possess more suitable mechanical strength for load bearing and drug delivery.

###### PLGA.

**FIGURE 6:**
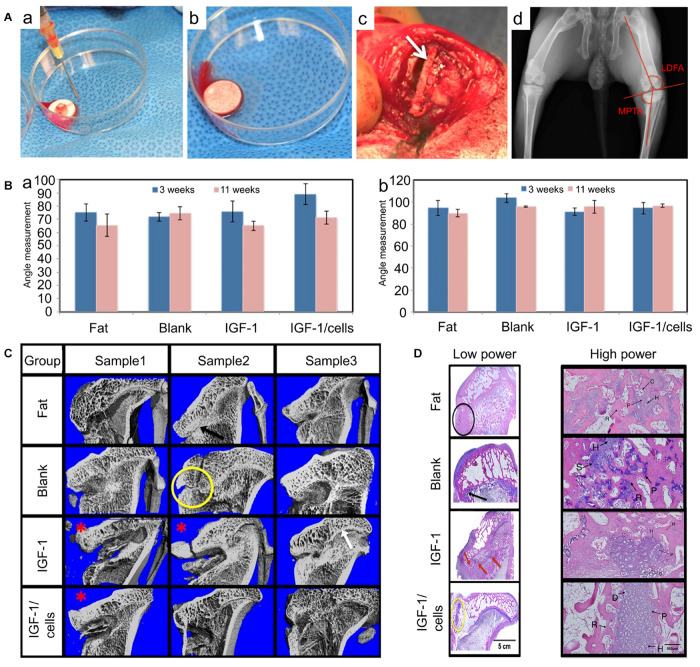
PLGA scaffolds in the treatment of GP injuries. **(A)** (a–c) The morphology of PLGA scaffolds *in vitro* and *in vivo.* (d) Measurements of the lateral distal femoral angle (LDFA) and the medial proximal tibial angle (MPTA). **(B)** (a) MPTA and (b) LDFA results of GP injuries at 3 weeks (without bone bridge resection) and 11 weeks (8 weeks after scaffolds implantation). **(C)** Micro-CT of the tibia in four treatment groups. **(D)** The histological results of GP repair with low power and high power in different groups (reproduced with permission from Clark et al., 2015).

PLGA is a promising synthetic polymer material suitable for the treatment of GP damage. It has alterable mechanical properties by controlling the proportion of lactic acid and glycolic acid, which makes it suitable for cartilage tissue implantation (Yan et al., 2017). When implanted *in vivo*, PLGA scaffolds will be degraded into lactic acid and glycolic acid which will be eliminated through GP metabolic pathway (Fathi-Achachelouei et al., 2020). Moreover, thanks to its controllable drug release kinetics, PLGA microspheres have also been approved for drug delivery by the Food and Drug Administration (FDA) (Zuo et al., 2016). Certainly, when utilized as a drug delivery vehicle of IGF-1, more chondrocytes and Col II were observed both *in vitro* and *in vivo* (Sundararaj et al., 2015). In a detailed study, Clark et al. (2015) used PLGA scaffolds alone or loaded with IGF-1 and cells for the treatment of GP injuries, PLGA alone had therapeutical effects as it showed more chondrocytes accumulation compared to fat grafts used in the clinic. Moreover, in our study BMSCs loaded with IGF-1 improved chondrocyte proliferation with more chondrocyte accumulation, and inhibited bone formation than scaffolds alone or IGF-1 delivered alone (Figure 6). All these results unravel that the PLGA scaffold is a good interpositional material and an appropriate carrier for GPs reconstruction.

###### Polylactic Acid.

Polylactic acid (PLA) is also a hydrophobic polyester used in biomedical applications (Yao et al., 2020). As a semicrystalline polymer, its crystallinity is approximately 37%, its glass transition temperature is approximately 67°C, and its melting temperature is approximately 180°C (Wan and Zhang, 2018). In addition, PLA is a thermoplastic polymer with high mechanical strength and low degradation rate (Georgiopoulos et al., 2018). Similar to PLGA, PLA is degraded in the form of oligomers, and its degradation products are lactic acid which is present in human body and can be metabolized *via* natural pathways (Gremare et al., 2018). The disadvantages of PLA are its poor thermal stability, high hydrophobicity, and brittleness (Vroman and Tighzert, 2009; Saini et al., 2016). However, there are only a few studies available using PLA scaffolds in the treatment of GP injuries. Implanted in proximal tibial GP defects in rabbits, the treatment of PLA scaffolds combined with chondrocytes resulted in new columnar chondrocytes formation, indicating that PLA is an appropriate material to fabricate scaffolds in GP related cartilage tissue engineering (Zhou et al., 2000, 2003).

##### Composite Materials

Scaffolds made by composite materials are currently increasingly used. Given the disadvantages of single materials, it is reasonable to mix materials with complementary advantages together when fabricating a scaffold (Keplinger et al., 2019; Trakoolwannachai et al., 2019). As described above, the remarkable ECM material derived from GP is a typical composite material mainly composed of GAGs and Col II (Horton et al., 2020). With tunable mechanical properties and excellent biocompatibility, composite materials can suitably simulate the internal environment and exhibit good cell affinity. For example, agarose hydrogel is a biocompatible material with poor mechanical properties, and is rapidly degraded *in vivo* (Zarrintaj et al., 2018). To overcome these limitations, agarose based composite materials are necessary. Kumar et al. (2018) synthesized a composite material with agarose and chitosan, and this new material-based scaffolds showed more suitable degradation rates and higher mechanical strength which may be more suitable for cartilage tissue engineering. Not only do natural composite materials have better characteristics, but also composite materials made by natural and synthetic materials are advantageous. For example, Wang et al. (2016) utilized a composite scaffold synthetized by PLGA, Col, and silk fibroin for cartilage repair, by altering the ratio of constituents. The *in vitro* studies indicated this composite scaffold promoted MSCs proliferation and differentiation without side effects, and the *in vivo* results showed enhanced cartilage regeneration in cartilage defects (Wang et al., 2016). In another study treating GP arrest in pigs, a scaffold consisting of chitosan and Col I was investigated. The composite material was more stable in the simulated body environment and the mechanical properties were significantly better than the single materials (Planka et al., 2012). In conclusion, scaffolds made by composite materials will be a promising solution for GP repair and regeneration (Table 1).

**TABLE 1:** Recently published experimental studies.

References	Seed cells	Growth factors	Scaffolds	Technique	Animal models	Results
Li et al. (2017)	BMSCs		ECM	New freeze-drying technique	Rabbits	Reduced the angular deformity and length discrepancy, observed neogenetic GPs
Gultekin et al. (2020)	BMSCs or Chondrocytes		Cell sheets		Rabbits	Prevented endochondral ossification, promoted bone growth
Lee et al. (2016)	Chondrocytes		Cell synthesized ECM	Cell culture	Rabbits	Minimized the deformity of rabbits
Tomaszewski et al. (2016)	Chondrocytes		A cartilago-fibrinous construct		Rabbits	Satisfactory graft integration and fair restitution of GP architecture
Sundararaj et al. (2015)		IGF-1	PLGA	The salt leaching technique	Rabbits	Observed neogenetic cartilage
Clark et al. (2015)	BMSCs	IGF-1	PLGA	The salt leaching technique	Rabbits	Increased chondrocyte density and inhibited bone bridge formation
Azarpira et al. (2015)	BMSCs		Chitosan	The freeze-drying technique	Albino rabbits	Less angular deformity with more MSCs concentration
Erickson et al. (2020)			Alginate/chitosan hydrogels		Rats	50:50 of irradiated alginate and chitosan produced the most cartilage tissue
Erickson et al. (2017)			A chitosan microgel		Rats	Neogenetic cartilage was observed
Lee D. et al. (2019)	Chondrocytes		Allogeneic decalcified bone matrix		Rabbits	Prevent limb deformity
Sananta et al. (2020)	Adipose-derived cells		Bone wax		Rat	Prevented bone bridge formation

## The Potential Applications of 3D Printing

### Utilization of 3D Printing in Cartilage Tissue Engineering

Over the last two decades, the technology of 3D printing has played a crucial role in the development of cartilage tissue engineering. A significant breakthrough has involved the utilization of biomimetic materials. As described above, ECM is an excellent material that provides a suitable microenvironment for chondrocytes growth and proliferation. Recently, several studies have explored its strengths for fabricating scaffolds. After removing all the cellular materials the ECM can be obtained from fat, cartilage, heart, or muscle tissue. The decellularized ECM has successfully been transformed into bioink for 3D printing (Fahimipour et al., 2019). Pati et al. (2014) produced 3D printed scaffolds with a porous 3D structure using cartilage derived ECM with a PCL framework. When cultured with MSCs, high cell viability and cartilage-related gene expression were observed, suggesting the 3D printed scaffolds would provide critical stimuli for MSCs growth, engraftment, and long-term functions (Pati et al., 2014). Another study printed an alginate reinforced ECM scaffold for cartilage regeneration, the ECM based scaffold was capable of supporting MSCs and could deliver growth factors to promote robust chondrogenesis with a high level of Col II expression (Rathan et al., 2019). A different method used to achieve biomimetic materials involves using composite materials. Many studies have prepared cartilage imitating materials with chitosan-PCL/silk firoin composite (Thunsiri et al., 2020), chitosan-gelatin hydrogel/PLGA composite (Schneider et al., 2018), or chitosan/gelatin/sodium β-glycerophosphate composite (Hu et al., 2019). Scaffolds printed by these chondro-inductive materials have been shown to stimulate chondrocytes survival and proliferation (Muller et al., 2017).

Thanks to the accurate control of 3D printing, another breakthrough achieved is the production of gradient scaffolds with distinct regions (Daly et al., 2017). Because gradient structures are present in human cartilage, it is reasonable to used scaffolds with various pore sizes. The heterogeneous interspace has a direct impact on nutrient distribution, which determines the chondrogenesis (Li et al., 2020). Through 3D fiber deposition technology, Di Luca et al. (2016) prepared a gradient scaffold with four distinct pore sizes of 326, 540, 744, and 968 μm. MSCs seeded in the gradient scaffold observed enhanced chondrogenesis with more GAG deposition compared with non-gradient scaffolds (Di Luca et al., 2016). Therefore, scaffolds with gradient structure are considered as a good strategy to promote chondrogenic differentiation of MSCs.

### Potential Superiorities of 3D Printing in GP Repair

Since the GP has three distinct zones with diverse differentiation stages of chondrocytes and different ECM components, the mechanical properties of each region are also distinctive (Shaw et al., 2018). From this standpoint, a 3D printing technique will be a fitting method as it produces scaffolds with gradient pore sizes possessing heterogeneous mechanical strength (Montazerian et al., 2019). Moreover, when printing different regions of a scaffold, using bioink with distinct strengths will also change the mechanical properties in different parts of the scaffold, which will best imitate the physiological structure of the GP.

Furthermore, chondrocytes with gradient density in scaffolds could be achieved by 3D bioprinting technology. Ren et al. (2016) bioprinted a cell gradient scaffold using Col II and chondrocytes and cultured it for several weeks. The gradient chondrocyte density resulted in a gradient deposition of ECM, making it possible to achieve the distinct GP ECM through variable cell density bioprinting (Ren et al., 2016).

Previous investigations have also suggested that more scaffolds improve the restore the physical structure, the better enhance of cartilage regeneration (Pati et al., 2014; Di Luca et al., 2016). Therefore, we expect the 3D printing technology will be applied widely in cartilage tissue engineering to treat GP injuries.

## Future Directions

Due to the avascular and hypoxic characteristics of cartilage tissue, healing is difficult after a severe injury (Wang D. Q. et al., 2020). Based on this shortcoming, cell-based cartilage tissue engineering technology has been proposed for the treatment of cartilage damage. In this cartilage repair system, seed cells are provided directly, and scaffolds made of biomaterials are used as carriers to fill the defects (Munir et al., 2020). Inspired by this system, the same method can be applied to GP injuries because of the limited self-healing capacity. Tissue engineered GPs have been explored in many studies; however, many problems remain unresolved as well.

### Mechanism of GP Development

At present, the precise regulatory mechanism of the GP is not clear. It is critical to unravel how different cytokines interact to precisely regulate the growth of long bones. In the future, a deeper understanding of the regulatory signaling pathways in skeletal development will make it possible to inhibit bone bridge formation with medicines that modulate specific signaling pathways. Moreover, additional studies are needed to clarify the specific phenotype of GP cells. Differences between chondrocytes in the GPs and chondrocytes in articular cartilage should also be elucidated. Recently, stem cells in the resting zone have been identified (Newton et al., 2019), but how these cells proliferate and differentiate into hypertrophic chondrocytes, and what determines the fate of hypertrophic chondrocytes still remains unknown. The essential physiology of GP chondrocytes needs to be further deepened.

### Repair Cells

Recently, much progress has been achieved in the area of tissue engineered cartilage repair in GP injuries. The most extensively used seed cells are BMSCs and chondrocytes. However, other stem cells like embryonic stem cells and induced pluripotent stem cells (iPSCs) are also frequently used in cartilage tissue engineering. Thus, it is worth investigating the application of other types of stem cells in the treatment of GP injuries. Given the proficient application of iPSCs in cartilage repair system, it would be easier to apply iPSCs for the treatment of GP injuries in the future. Thanks to the easy accessibility and multiple differentiation potentials, iPSCs will likely play a critical role in clinical applications (Swaroop et al., 2018). In addition, due to the current limitations in basic research, the methods used to identify regenerated GP chondrocytes relies on histological observation and immunological detection of cartilage-related proteins (Fernandez-Iglesias et al., 2020). Nonetheless, these methods can only evaluate chondrocytes in general, while the identification of GP-specific chondrocytes still remains an unsolved problem. It is expected that in the future, we can find a specific protein related to the GP chondrocytes in order to accurately identify GP reconstruction.

### Local Bioactive Microenvironment

At present, a self-healing system of tissue engineered GPs is widely used for damaged GPs. Experimental results show that cartilage repair is successful, a large number of chondrocytes are regenerated, and cartilaginous ECM is synthesized in large quantities (Hong Y. P. et al., 2020). Following bone bridge resection, defects can be filled with scaffolds, which are loaded with seed cells (Erickson et al., 2018). If necessary, growth factors could also be added to enrich the regeneration microenvironment (Sundararaj et al., 2015). As for the selection of growth factors, during the process of development to maturity, there are a variety of regulatory factors involved in GP regulation, such as Ihh, PTHrP, and BMP-2 (Hallett et al., 2019). But unfortunately, the application of these bioactive substances has not been fully explored. Future studies should assess the therapeutic effects of these active factors.

### Structure Design of Scaffolds

The scaffold materials used currently have many shortcomings. Considering the numerous composite materials available for cartilage tissue engineering exhibiting excellent performance, 3D printing technology will allow to achieve a precise control in fabrication technology, which has been widely used to produce scaffolds with complex and gradient structures (Hong H. et al., 2020). It is foreseeable that bionic scaffolds fabricated by composite materials will play an important role in treatment of GP injuries in the near future. Currently, scaffolds with various structures are used in cartilage tissue engineering, for example, to generate pores of different shapes and orientations, such as in grid, triangular, rectangular, or circular. Further, porous scaffolds having different porosity and pore sizes, with interconnected or unconnected pores, have all been applied to cartilage tissue engineering, but there is still no consensus on the best structure. Additional studies will be required to identify the most suitable parameters for GP reconstruction.

## Conclusion

The GP plays an important role in the longitudinal growth of long bones. Considering damage to GPs will result in limb length discrepancies and angular deformities, it is critical to identify more effective ways to address these problems. Inspired by the advances in the cartilage repair system, tissue engineered GPs have received greater attention as a potential therapy for GP regeneration. The construction of the implants theoretically should include repair seed cells for cartilaginous tissue reconstruction, growth factors to induce chondrogenesis, as well as scaffolds for load bearing, active substance delivery, and enhancing the regenerative microenvironment. This review mainly focused on the developments of tissue engineered GPs in the treatment of GP injuries. The unsolved problems and challenges that impede its clinical application were unraveled. Moreover, combined with the advantages of 3D bioprinting technology to fabricate scaffolds with gradient bionic structure, tissue engineered GPs will help overcome the challenges in the treatment of GP injuries in the future.

## Author Contributions

XW and ZL conceived the study and wrote the manuscript. CW and HB carried out the analysis with the help of ZW, YL, and YB. MR, HL, and JW contributed intellectually throughout the study. All of the authors critically reviewed this manuscript and approved the final draft.

## Conflict of Interest

The authors declare that the research was conducted in the absence of any commercial or financial relationships that could be construed as a potential conflict of interest.
